# Expression of HNF4alpha in the human and rat choroid plexus – Implications for drug transport across the blood-cerebrospinal-fluid (CSF) barrier

**DOI:** 10.1186/1471-2199-10-68

**Published:** 2009-07-03

**Authors:** Monika Niehof, Jürgen Borlak

**Affiliations:** 1 Fraunhofer Institute for Toxicology and Experimental Medicine, Center of Molecular Medicine and Medical Biotechnology, Nikolai-Fuchs-Str. 1, 30625 Hannover, Germany; 2 Center of Pharmacology and Toxicology, Medical School of Hannover, Carl-Neuberg-Str. 1, 30625 Hannover, Germany

## Abstract

**Background:**

The choroid plexus consists of highly differentiated epithelium and functions as a barrier at the interface of the blood-cerebrospinal-fluid (CSF). This tissue may therefore determine the bioavailability and transport of drugs to the brain. Little is known about the expression of drug and xenobiotic metabolizing enzymes (DME) and of drug transporters in the human choroid plexus. Notably, the transcription factor and zinc finger protein HNF4alpha is a master regulator of DMEs and of drug transporters. As of today its activity in the blood-CSF barrier is unknown. Here we report our efforts in determining HNF4alpha activity in the regulation of ABC transporters in the human and rat choroid plexus.

**Results:**

We report expression of HNF4alpha by qRT-PCR and by immunohistochemistry and evidence transcript expression of the ATP-binding cassette transporters ABCB1, ABCB4, ABCC1-6 in choroid plexus. Additionally, HNF4alpha DNA binding activity at regulatory sequences of ABCB4 and ABCC1 was determined by EMSA bandshift assays with a specific antibody. We then performed siRNA mediated functional knock down of HNF4alpha in Caco-2 cells and found ABCC1 gene expression to be repressed in cell culture experiments.

**Conclusion:**

Our study evidences activity of HNF4alpha in human and rat choroid plexus. This transcription factor targets DMEs and drug transporters and may well determine availability of drugs at the blood-CSF barrier.

## Background

Drug delivery to the brain is mediated by several factors, most notably transport across the blood brain (BB) and the choroid plexus barrier; the latter displays drug-metabolizing enzyme and drug transport activity. It may therefore determine the overall cerebral bioavailability of drugs [1]. Specifically, the choroid plexus is located within brain vesicles. It is composed of a tight monolayer of polarized epithelial cells and forms the blood-cerebrospinal-fluid (CSF) barrier. Together with the blood-brain barrier, formed by endothelial cells of brain capillaries, it functions as the main interface between the central nervous system (CNS) and the peripheral circulation. Within the CNS this tissue is of great pharmacological interest, but information on the expression of efflux transporters such as the ATP binding cassette (ABC) proteins is missing [2]. In contrast, their expression in liver, kidney, and intestine has been studied in considerable detail [3-5]. Indeed, the ABC drug transporters extrude a variety of structurally diverse drugs, drug conjugates and metabolites in an active, ATP-dependent manner and even against high concentration gradients. The three main ABC families considered to be involved in the disposition of xenobiotics include the ABCB family (MDR subfamily, multidrug resistance, e.g. ABCB1/MDR1), the ABCC family of multidrug resistance proteins (MRP subfamily, multidrug resistance related proteins, e.g. ABCC2/MRP2), and the breast cancer resistance protein (ABCG2/BCRP) of the ABCG family [3,4]. However, comprehensive studies on the expression levels of ATP transporters in the human choroid plexus have not been attempted.

Notably, there is clear evidence for HNF4α to play a role in the transcriptional control of drug transporters. Specifically, HNF4α is a member of the nuclear receptor superfamily and one of the key players in liver biology [6-8]. Among the genes regulated by HNF4α are a broad range of xenobiotic-metabolizing cytochrome P450 isozymes [9,10], UDP-glucuronosyltransferases [11], sulfotransferases [12] and transporters including organic anion transporter 2 [13], organic cation transporter 1 [14], the ABC transporter *ABCC2 *[15], *ABCC6 *[16], *ABCG5 *[17] and *ABCG8 *[17]. Although there is clear evidence for HNF4α to be of key importance in the control of drug metabolism it may also play a role in the regulation of transporters in the choroid plexus. Here we report our efforts in mapping HNF4α to human and rat choroid plexus. We determined HNF4α DNA binding activity and searched for transcript expression of various *ABCB *and *ABCC *transporters in the human choroid plexus. Apart from qRT-PCR and immunohistochemistry studies we evidence *ABCC1 *gene expression to be highly dependent on HNF4α as determined in functional knock down studies. Overall, we provide evidence for HNF4α to be an important regulator of ABC drug transporters in the choroid plexus and thus may impact efficacy of pharmacotherapy targeted to the brain.

## Results

**Figure 1:**
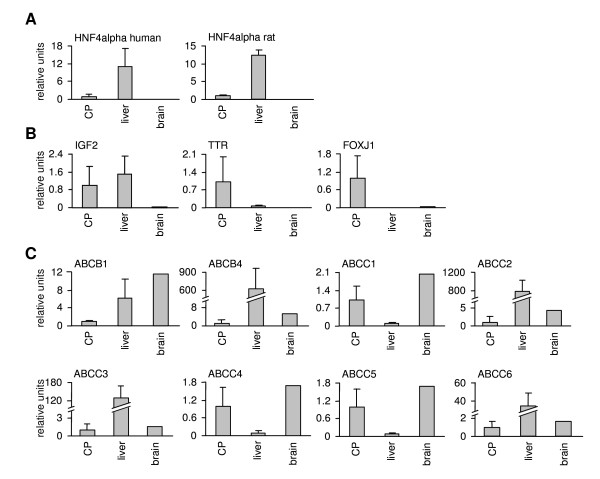
**Gene expression of HNF4α and different ABC transporters in the choroid plexus**. Gene expression of HNF4α and different ABC transporters in the choroid plexus. A: HNF4α gene expression in human (n = 3) and rat choroid plexus (n = 3) was measured by quantitative real-time RT-PCR and determined relative to expression of mitATPase6, which served as a housekeeping gene. Expression levels were compared to liver and total brain. Mean values + SD are shown. B and C: Gene expression of FOXJ1, IGF2 and TTR (B) and of different members of the ABCB and ABCC family (C) was analyzed in human choroid plexus by quantitative real-time RT-PCR and determined relative to expression of mitATPase6, which served as a housekeeping gene. Expression levels were compared to liver (n = 4) and total brain. Mean values + SD are shown.

**Figure 2:**
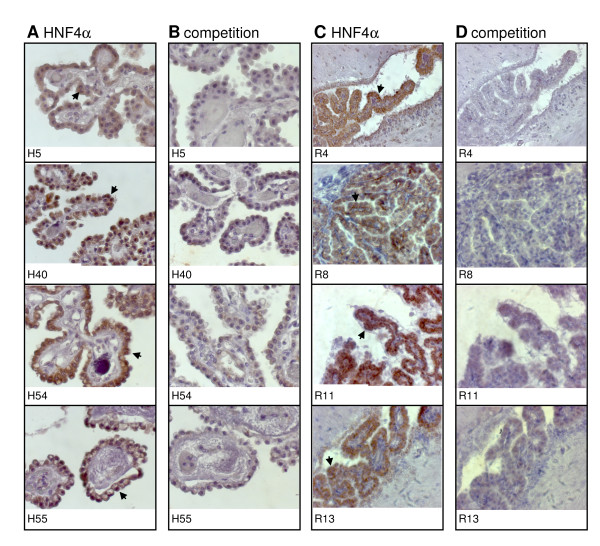
**Immunohistochemical detection of HNF4α in the choroid plexus**. Slices of human (A, B) and rat (C, D) choroid plexus probes were stained with polyclonal antibodies against HNF4α. Arrows indicate representative HNF4α positive cells (A, C). To confirm specificity of the immunohistochemical localization antibodies were preabsorbed with excess of antigens for HNF4α (B, D). Patient identification numbers were indicated respectively and patient characteristics are given in Table 5. Magnification ×400.

Initially, we searched for *HNF4α *transcripts in individual samples of human and rat choroid plexus and confirmed gene expression of *HNF4α *by quantitative real time RT-PCR (Figures 1A). We found *HNF4α *transcript expression in human and rat choroid plexus to account for approximately a tenth of its expression in the liver (Figures 1A). It is of considerable importance that *HNF4α *expression in the human and rat choroid plexus is restricted to P1 promoter driven isoforms (Table 1). Furthermore, we studied expression of the insulin-like growth factor 2 (*IGF2*), transthyretin (*TTR*) and the transcription factor *FOXJ1 *to further qualify choroidal epithelial cells of the brain [18,19]. These transcripts are specifically enriched in choroid plexus. We observed abundant expression of *IGF2*, *TTR *and *FOXJ1 *in human choroid plexus as compared to total brain RNA extracts (Figures 1B). There is the need to study histological well qualified tissue, as studies with total brain RNA extracts would render findings meaningless as will be discussed later on. Unfortunately, sufficient human choroid plexus tissue suitable for the harvest of nuclear protein and to perform western blotting as well as EMSA assay could not be obtained. We nonetheless demonstrate HNF4α protein expression by immunohistochemistry by use of a specific HNF4α antibody for human (Figures 2A) and rat choroid plexus (Figures 2C). To confirm specificity an excess of antigen preabsorbed to the antibody was used (Figures 2B, D).

**Table 1:** HNF4α isoform expression in human and rat choroid plexus.

**Patient**	**HNF4αP1**	**HNF4αP2**
Patient 100-04	0.0342	0
patient 44-04	0.0025	0
patient 62-04	0.4110	0

**Animal**	**HNF4αP1**	**HNF4αP2**

rat 1	0.097	0
rat 2	0.348	0
rat 3	0.158	0

HNF4αP1 and P2 gene expression was measured in human and rat choroid plexus (n = 3, respectively) by real-time quantitative RT-PCR and computed relative to expression of mitATPase6, which served as a housekeeping gene. Patient characteristics are given in Table 5. Caco-2 cells and pancreatic tissue served as controls for HNF4αP2 expression in human and rats, respectively.

We then analyzed expression of different members of the ABCB (MDR subfamily, multidrug resistance)/ABCC (MRP subfamily, multidrug resistance related) gene families in the human choroid plexus by quantitative real time RT-PCR and report results for n = 3 individual human choroid plexus samples. mRNA expression of *ABCB4 (MDR2/3), ABCC1 (MRP1), ABCC2 (MRP2)*, *ABCC3 (MRP3), ABCC4 (MRP4), ABCC5 (MRP5) *and *ABCC6 (MRP6) *was comparable to its expression level in commercially available control total human brain RNA extracts (Figures 1C). mRNA expression of *ABCB1 (MDR1) *determined in choroidal epithelium was lower than in human liver (n = 4) and in brain (Figures 1C).

**Figure 3:**
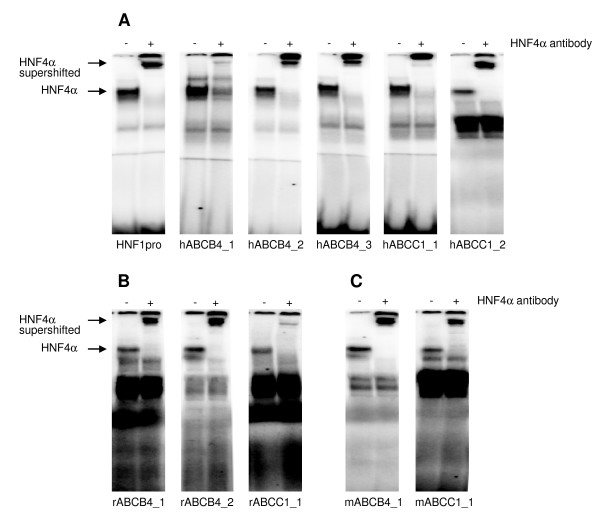
**HNF4α binding to promoters of ABCB4 and ABCC1**. Electrophoretic mobility shift assays with 2,5 μg Caco-2 cell nuclear extract and oligonucleotides corresponding to the A-site of the HNF1α promoter (HNF1pro) and to putative HNF4α binding-sites within human (A), rat (B) and mouse (C) ABCB4 and ABCC1 as ^32^P labeled probe. In supershift assays an antibody directed against HNF4α (+) was added. For oligonucleotide sequence information see Table 2 and 3.

We then searched for HNF4α binding sites in proximal promoter sequences (up to -7000 bp) of drug transporter coding genes. For this purpose, we used two different bioinformatic approaches (see Material and Methods section for a description of the employed algorithms). We observed three binding sites within the *ABCB4 *promoter, spaced approximately by 600 bp and 1600 bp and two recognition sites within the *ABCC1 *promoter (Table 2). Predicted binding sites were confirmed by EMSA band shift assays. We used ^32^P-labeled double-stranded DNA probes to specifically probe for HNF4α-sites located in the human *ABCB4 *(hABCB4_1, hABCB4_2 and hABCB4_3) and in the human *ABCC1 *(hABCC1_1, hABCC1_2) gene. Note, DNA binding of nuclear extracts to the A-site of the *HNF1α*-promoter (HNF1pro) served as a positive control. This site is an established recognition site for HNF4α. Unfortunately, sufficient amount of human choroid plexus suitable for the isolation of nuclear protein could not be obtained. Instead, we used nuclear extracts isolated from the human intestinal cell line Caco-2 which expresses several ABC transporter genes [20] and therefore is a rich source of HNF4α nuclear protein. Indeed, HNF4α protein expression in Caco-2 cells is comparable to organs such as the liver [21]. As depicted in Figures 3A we observed strong binding of HNF4α to the A-site of the *HNF1α*-promoter. We also observed strong binding of HNF4α to the predicted sites in the promoters of *ABCB4 *and *ABCC1 *(Figures 3A); bands could be shifted with a specific HNF4α antibody therefore demonstrating selectivity and specificity of the assay. Alignment of human, rat and mouse *ABCB4 *and *ABCC1 *genes did not identify common HNF4alpha binding sites. This points to differences in the molecular organization of ABC promoters in orthologous genes. HNF4alpha binding sites for the rat and mouse *ABCB4 *and *ABCC1 *genes are given in Table 2, whereas the sequences of oligonucleotides to confirm the predicted sites experimentally are shown in Table 3. As shown in Figures 3B and 3C EMSA band shift assays confirmed binding of HNF4α to rat and mouse *ABCB4 *and *ABCC1 *targeted sequences.

**Table 2:** Predicted binding sites for HNF4α in ABC genes.

**Gene name**	**Accession number**	**bp relative to transcription start site**	**Score core/matrix**	
**Human**				
ABCB4	NM_000443.3	-6274 to -6252	V$zemlin13_11045	1.000/0.896
		-4680 to -4658	V$HNF4_01	1.000/0.990
			V$zemlin13_1104	1.000/0.997
		-4063 to -4041	V$HNF4_01	1.000/0.954
			V$zemlin13_11045	1.000/0.958
ABCC1	NM_004996.3	-1756 to -1734	V$HNF4_01	0.915/0.925
		-5266 to -5244	V$HNF4_01	1.000/0.944
			V$zemlin13_11045	1.000/0.969

**Rat**				
ABCB4	NM_012690.1	-4584 to -4562	V$zemlin13_11045	1.000/0.894
		-39 to -17	V$HNF4_01	1.000/0.943
			V$zemlin13_11045	1.000/0.978
ABCC1	NM_022281.2	-2146 to -2124	V$HNF4_01	1.000/0.908

**Mouse**				
ABCB4	NM_008830.2	-5281 to -5259	V$zemlin13_11045	1.000/0.907
ABCC1	NM_008576.2	+2626 to +2648 (Intron 1)	V$HNF4_01	1.000/0.924
			V$zemlin13_11045	1.000/0.910

Transfac matrix (V$HNF4_01) or self-generated matrix (V$zemlin13_11045) with a setting of core and matrix similarity 0.9 was used.

**Table 3:** Shift-probes sequences.

**Gene**	**Oligo name**	**Sequence**
**Human**		
HNF1α	HNF1pro	AGGGCTGA **AGTCCA A AGTTCA **GTCCCTTC
ABCB4	hABCB4_1 (GS49)	AGAA **AGGTCA A AGGCAA **AGCA
	hABCB4_2 (GS50)	GTGC **AGGGCA A AGGTCA **GATT
	hABCB4_3 (GS51)	GCTA **AGTCCA A AGATCA **ATTC
ABCC1	hABCC1_1 (GS48)	TTGAC **AGTATA A AGGTCA **AGCAA
	hABCC1_2 (GS171)	TTCAT **AGAGCA A AGTACA **GCAAC

**Rat**		
ABCB4	rABCB4_1 (GS166)	AGAAG **CCCTCA A AGTCCA **CCTAT
	rABCB4_2 (GS167)	CGCAG **CGAGCA A AGTCCA **GGTCT
ABCC1	rABCC1_1 (GS169)	GACAC **GGGCCA A AGACCC **ACAGA

**Mouse**		
ABCB4	mABCB4_1 (GS168)	ATAGC **CATGCA A AGTCCA **AAAGA
ABCC1	mABCC1_1 (GS170)	TCCCT **TGTGCA A AGGTCT **AGGAT

The central five nucleotides of the HNF4α binding site are highlighted in bold letters. The two direct repeats (DR1) separated by a spacer of one nucleotide are underlined.

To further probe for the role of HNF4α in ABC gene regulation we employed an siRNA approach. Specifically, siRNA-mediated functional knock down of HNF4α in the human Caco-2 cell line resulted in significantly decreased gene expression of *ABCC1 *(Table 4; for transfection efficiency and stable expression of *mitATPase6 *after transfection see Niehof and Borlak, 2008 [22]). These results confirm *ABCC1 *to be a gene target of HNF4α. *ABCB4 *expression in Caco-2 cells is near the limit of detection. Consequently, knockdown experiments are not meaningful.

**Table 4:** Functional knock down of HNF4α in cultures of human Caco-2 cells.

**Gene**	**Gene expression 48 h after HNF4α siRNA transfection [%]**	p-value*
HNF4α	24.0	0.0031

ABCC1	56.4	0.0478

Functional knock down of HNF4α was already reported [22]. * A student's t test was done to compare HNF4α siRNA transfection against negative siRNA transfected controls. The results were considered significant when the p value was less than 0.05. Gene expression was determined by real-time quantitaive RT-PCR in triplicates; mitATPase6 served as housekeeping gene. The ΔΔC_T_-method was used to determine repression of gene expression. Values for HNF4α siRNA transfection represent transcript abundance relative to negative siRNA transfected controls, which were set to 100%.

## Discussion

Our study aimed for a better understanding of the role of HNF4α in the regulation of drug transporters. Here we present evidence for expression of HNF4α in the epithelium of the CSF barrier. By applying position weight matrices to genomic sequences of ABC transporters we were able to predict HNF4α binding sites in the promoters of the *ABCB4 *and *ABCC1 *gene. The predicted binding sites were then confirmed by EMSA band shift assays. We propose a role for HNF4α in the regulation of drug transporters of the choroid plexus. Notably, HNF4α is a key player in the regulation of genes coding for various metabolic pathways and of xenobiotic metabolism [6-9,23,24]. This protein also promotes expression of an epithelial phenotype [25-27]. Specifically, epithelium of the choroid plexus cells is highly differentiated and functions as a blood-CSF barrier. The expression of HNF4α in the epithelium of the choroid plexus and its DNA binding to regulatory sequences of drug transporters is a novel finding. Its expression accounts for approximately a tenth of HNF4α expression found in liver. Furthermore, there is evidence for HNF4α to undergo alternative splicing with isoforms may arising from alternative splicing and/or usage of two different promoters [6]. Specifically, the P1 promoter generates six different isoforms (HNF4α1-α6); but activation of the P2 promoter results in isoforms HNF4α7-α9. P2 promoter-driven HNF4α isoforms are expressed throughout mouse liver development, but disappear after birth, while P1 promoter-driven transcripts are abundantly expressed postnataly [28]. Additionally, P2 isoforms are induced in mouse and human hepatocellular carcinoma [29,30] and are primarily expressed in human pancreatic islets and exocrine cells [31]. In the case of the choroid plexus it appears that expression of HNF4α is restricted to P1 promoter-driven isoforms. HNF4α protein expression in human and rat choroid plexus could be clearly evidenced by immunohistochemistry. To further qualify tissue preparations of choroid plexus expression of *IGF2, TTR *and *FOXJ1 *was investigated; their expression are accepted genetic markers of the choroid plexus [18,19]. We validated tissue preparations by morphological and genetic markers. Unlike previous studies where expression of *HNF4α *transcripts could not be evidenced in total RNA extracts of the brain [6], we were able to confirm expression of *HNF4α *in the choroid plexus of human and rat brain. Most certainly, total brain RNA extracts dilute copy number of *HNF4α *transcripts to presumable levels below the limit of detection. Here, we evidence binding of HNF4α to regulatory sequences of drug transporters expressed in the choroid plexus and analyzed gene expression of ABC transporters in patients with different causes of death, but the functional significance of the newly identified HNF4α binding sites in activating the ABCB4 and ABCC1 promoters still needs to be established.

In the past attempts to detect HNF4α DNA binding in a choroid plexus papilloma failed [32]. The investigators performed EMSA experiments with nuclear extracts of rat liver, kidney and intestine and of SV40-induced choroid plexus papilloma of transgenic mice, but unfortunately probed for HNF4alpha binding with an oligonucleotide corresponding to the HNF4α binding site in the mouse TTR (transthyretin) promoter. The authors did not employ an antibody in EMSA band shift assays; instead, competition with excess of unlabeled probes was done. Although the authors described a weak binding of HNF4α with nuclear extracts from kidney they considered intestine as well as choroid plexus as deficient for HNF4α binding. There is a need to consider tissue specific DNA binding activity. HNF4α binding at the TTR promoter is much less as compared to the A-site in the HNF1 promoter (not exceeding a tenth, data not shown). As detailed above, HNF4α gene expression in human and rat choroid plexus is approximately one tenth of its expression in the liver (see Figures 1A). It is therefore not surprising that previous investigators [32] failed to detect HNF4α protein in intestine and choroid plexus, even though the authors described weak expression of this protein in kidney. By now, it is well established that HNF4α expression is not restricted to liver, but also functions in kidney and intestine [33,34]. Here we evidence by immunohistochemical staining HNF4α to be expressed in epithelium of human and rat choroid plexus (see Figures 2).

Notably, the choroid plexus functions as a barrier for drug uptake to the brain. This tissue expresses drug metabolizing enzymes (DMEs) and some transporters [1]. Expression of DMEs in the choroid plexus is part of a defense program to prevent entry of xenobiotics into the brain. The blood-CSF barrier also regulates entry and distribution of various pharmacologically active compounds between the blood and the CSF interface and is basically involved in numerous exchange processes thereby determining the supply of the brain with nutrients and hormones [1]. Indeed, endogenous metabolites, as well as neurotransmitter and metabolites from the brain are cleared via this barrier [19]. In drug therapies efflux transporters of the ABC-family are of pivotal importance in determining therapeutic tissue levels. In the past research focused on their regulation in liver, kidney and intestine [3,4]. The knowledge on drug transporter in specialized tissues of the brain is incomplete. Specifically, ABCB (MDR) proteins accept a broad range of substrates and may transport large lipophilic, neutral or cationic compounds. This includes a vast number of neuropharmacological drugs such as antiepileptic and antiviral drugs, antidepressants, opiods, antipsychotics and tranquilizer [35-40]. *ABCB1*, a transporter with highest expression in the gastrointestinal tract [4,41], is expressed at low levels in human choroid plexus, that is much lower than in liver and total brain. Our findings are consistent with results reported previously for rats [42]. Its apical expression in rat, mouse and human choroidal epithelium was shown previously [43]. Furthermore, *ABCB4 *is highly expressed in the liver, where it is acting as a "flippase" in transporting phospholipids into the bile, but ABCB4 can also bind and transport a subset of ABCB1 substrates with an overlap in substrate specificity [44]. As reported for *ABCB1, ABCB4 *is expressed at low levels in human choroid plexus, i.e. more than 500 fold lower than in liver. Low *ABCB4 *expression was also evidenced in choroidal epithelium of the rat [42], but to the best of our knowledge our study is the first report on expression in human choroid plexus. An apical distribution of ABCB1 in neonatal cultured rat choroid plexus cells imply a drug transport from blood into CSF [43]. In endothelial cells of small blood capillaries of the blood-brain barrier, apical located ABCB1 pumps drugs back into the blood stream and therefore limits drug penetration to the brain [2].

Likewise, the ABCC (MRP) proteins are multispecific organic anion transporters and accept glucurono-, glutathione- and sulfo-conjugates. They transport physiological substrate conjugates as well as drug conjugates. Expression of *ABCC1-6 *mRNA transcripts was reported for rat choroid plexus [42] as was expression of *ABCC1 *for human choroid plexus [43,45,46]. *ABCC1, ABCC4 *and *ABCC5 *were expressed in human choroid plexus at least at 10 fold higher than in liver; whereas *ABCC2, ABCC3 *and *ABCC6 *were expressed up to 800 fold lower than liver. Similar results were reported for *ABCC *transporter expression in rat choroid plexus [42]. Notably, ABCC1 is localized at the basolateral membrane of choroid plexus [43,45,46], but Gazzin et al [46] described a major difference in the localization of ABCB1 and ABCC1 proteins between the blood-brain and the blood-CSF barrier with strongest expression of ABCC1 at the choroidal epithelium. Indeed, ABCC proteins contribute to the protective role of the choroid plexus and mediate basolateral efflux of conjugates resulting from choroidal drug metabolism into the blood. Although it is known that the choroid plexus is important in regulating the distribution of various pharmacologically active compounds between the blood and the CSF, the characterization of the involved human ABC transporters gives new insights into the function of the CSF barrier.

Furthermore, ABCBs (MDRs) and ABCCs (MRPs) are inducible transporters and are highly responsive to chemotherapeutics, carcinogens, inflammation, heat shock, hypoxia and irradiation [47]. They are regulated by a complex network of transcriptional cascades, such as by multiple ligand activated nuclear receptors like retinoid X receptor (RXR), farnesoid X receptor (FXR), constitutive androstane receptor (CAR) and the xenobiotic receptor pregnane X receptor (PXR) [47,48]. There is also evidence for the transcription factors AP-1, p53, Egr-1 and WT-1 to participate in their regulation with NF-Y, Sp1 and Sp3 being involved in the constitutive expression [47]. Recently, an upregulation of *ABCB1, ABCB4 *and *ABCC4 *transcripts was reported in human embryonic kidney cells that conditionally expressed wild-type HNF4α [33]. An important role of HNF4α in the transcriptional control of drug transporters was reported for human hepatocytes as determined by adenoviral HNF4α-siRNA mediated knockdown [49]. We also employed an siRNA mediated functional knockdown of HNF4α and found ABCC1 gene expression to be massively repressed. There is a need to improve an understanding of the mechanism by which transporters are regulated. This will impact the design of novel CNS therapeutics. Targeting transporters may thus be useful in achieving therapeutic tissue levels of CNS drugs.

## Conclusion

We report expression of HNF4α in choroid plexus of the human and rat brain. This factor might regulate expression of some ATP binding cassette transporters. Targeting of HNF4α may impact efficacy of pharmacotherapy of CNS drugs.

## Methods

### Human and rat tissue

A total of n = 7 human and n = 7 rat tissues were analyzed. Samples of human choroid plexus (n = 3, gene expression analysis) were kindly provided by T. Arendt (Department of Neuroanatomy, Paul-Flechsig-Institute, University of Leipzig, Germany). Paraffin-embedded slices of human choroid plexus for immunohistochemistry (n = 4) were kindly provided by C. Grothe (Institute of Neuroanatomy, Hannover Medical School, Hannover, Germany). Human liver tissue (gene expression analysis) was obtained from patients undergoing hepatic resections and were kindly provided by J. Klempnauer (Department of Visceral and Transplantation Surgery, Hannover Medical School, Hannover, Germany). Patient characteristics are given in Table 5. Control human brain RNA was purchased from BD Biosciences (Heidelberg, Germany).

**Table 5:** Patient characteristics.

**Liver**					
**Patient Identification**	**Sex**	**Age**	**Information**	**Tissue**	**Application**

P29	F	40	Colorectal liver metastasis	Healthy tissue from liver resection	Gene expression analysis, comparison to choroid plexus

P2	F	70	Hepatocellular carcinoma	Healthy tissue from liver resection	Gene expression analysis, comparison to choroid plexus

P3	F	57	Hepatocellular carcinoma	Healthy tissue from liver resection	Gene expression analysis, comparison to choroid plexus

P4	M	67	Hepatocellular carcinoma	Healthy tissue from liver resection	Gene expression analysis, comparison to choroid plexus

**Choroid Plexus**					

**Patient Identification**	**Sex**	**Age**	**Cause of death**	**Tissue**	**Application**

44-04	F	63	liver cirrhosis	38 h post mortem	Gene expression analysis

62-04	F	79	ovarial carcinoma	33 h post mortem	Gene expression analysis

100-04	M	74	Acute myocardial infarction	48 h post mortem	Gene expression analysis

H5	M	53	Circulatory failure, liver cirrhosis	Choroid plexus	Paraffin-embedded slices for immunohistochemistry

H40	M	32	Circulatory failure, endocarditis	Choroid plexus	Paraffin-embedded slices for immunohistochemistry

H54	M	59	Respiratory insufficiency, pneunomia	Choroid plexus	Paraffin-embedded slices for immunohistochemistry

H55	M	81	Circulatory failure, generalized artheriosclerosis	Choroid plexus	Paraffin-embedded slices for immunohistochemistry

Samples of rat choroid plexus (n = 3, Sprague Dawley rats, gene expression analysis) were kindly provided by H. Hilbig and K. Spanel-Borowski (Department of Anatomy, University of Leipzig, Germany). Samples of rat liver and brain (Sprague Dawley rats, gene expression analysis) were generated in-house. Paraffin-embedded slices of rat brain (Sprague Dawley rats) containing choroid plexus regions for immunohistochemistry (n = 4) were kindly provided by C. Grothe (Institute of Neuroanatomy, Hannover Medical School, Hannover, Germany).

### Quantitative real-time RT-PCR

Analysis of human samples: Three human choroid plexus samples and four human liver samples were analyzed separately and used for calculation of the mean and standard deviation. Analysis of rat samples: Three rat choroid plexus samples, three rat liver and three rat brain samples were analyzed separately and used for calculation of the mean and standard deviation. Total RNA from choroid plexus and liver was isolated using the RNeasy Mini Kit (Qiagen, Hilden, Germany) according to the manufacturers recommendations. Subsequently to RNA isolation, a DNase I digest was performed. 4 μg total RNA from each sample was used for reverse transcription (Omniscript Reverse Transcriptase, Qiagen, Hilden, Germany). Quantitative real-time RT-PCR measurement was done with the Lightcycler (Roche Diagnostics, Mannheim, Germany) with the following conditions: denaturation at 95°C, annealing at different temperatures for 8 sec, extension at 72°C for different times and detection of SYBR-Green I-fluorescence at different temperatures. Detailed primer specific conditions and oligonucleotide sequence information are given in Table 6. Relative quantification was performed using the "Fit Points Method" of the LightCycler3 Data Analysis Software version 3.5.28 (Roche Diagnostics, Mannheim, Germany) by comparing the sample values to a standard curve within the linear range of amplification. This comparison was performed during each LightCycler Run (for genes of interest as well as for the housekeeping gene, i.e. mitATPase6). The standardized sample values for each gene of interest were divided by the standardized values of the housekeeping gene. The slope of external standard curves are given in Table 6, indicating the PCR efficiency for each amplicon.

**Table 6:** Real-time PCR primer sequences and amplification settings.

**Gene**	**Accession number**	**Species**	**Primer sequence**	**Fragment length**	**Annealing**	**Extension**	**Fluorescence**	**Slope**
HNF4α	NM_000457	human	fwd:CTGCTCGGAGCCACCAAGAGATCCATG	371 bp	60°C	15 sec	88°C	-3.629
			rev: ATCATCTGCCAGGTGATGCTCTGCA					

HNF4αP1	NM_178849	human	fwd: GAATGCGACTCTCCAAAACC	339 bp	68°C	14 sec	88°C	-3.058
	NM_178850		rev: GGCACTGGTTCCTCTTGTCT					
	NM_000457							

HNF4αP2	NM_001030003	human	fwd: GGGCTCCAGTGGAGAGTTC	342 bp	68°C	14 sec	88°C	-2.648
			rev: CATAGCTTGACCTTCGAGTGC					

FOXJ1	NM_001454	human	fwd: CTACTCGTATGCCACGCTCA	183 bp	60°C	8 sec	86°C	-3.459
			rev: CGAGGCACTTTGATGAAGC					

IGF2	NM_000162	human	fwd: GGTGCTTCTCACCTTCTTGG	298 bp	68°C	12 sec	90°C	-2.724
			rev: GGGGTATCTGGGGAAGTTGT					

TTR	NM_000371	human	fwd: CAGAAAGGCTGCTGATGACA	261 bp	60°C	11 sec	86°C	-3.438
			rev: GCCGTGGTGGAATAGGAGTA					

ABCB1	NM_000927	human	fwd: AAAAAGATCAACTCGTAGGAGTG	161 bp	60°C	7 sec	81°C	-3.331
			rev: GCACAAAATACACCAACAA					

ABCB4	NM_000443	human	fwd: CCACAGCGAACTGATGAAGA	301 bp	68°C	13 sec	83°C	-3.826
			rev: CACGACAAAGTAGGGCCATT					

ABCC1	NM_004996	human	fwd: AGTCCCCACAGAAGGAGTGG	358 bp	60°C	15 sec	87°C	-3.639
			rev: TCCCCGACCGTGGAGGATTT					

ABCC2	NM_000392	human	fwd: CCGTATCAGGTTTGCCAGTT	312 bp	60°C	13 sec	84°C	-3.756
			rev: CAACAGCCACAATGTTGGTC					

ABCC3	NM_003786	human	fwd: CTCAATGTGGCAGACATCGG	177 bp	60°C	8 sec	87°C	-3.731
			rev: GGGAGCTCACAAACGTGTGC					

ABCC4	NM_005845	human	fwd CTTGGAGAGGAGTTGCAAGG	236 bp	68°C	10 sec	78°C	-4.479
	NM_001105515		rev: GCTGTGTTCAAAGCCACAGA					

ABCC5	NM_005688	human	fwd: CCTGTTTGGGAAGGAATATGA	203 bp	60°C	9 sec	88°C	-3.446
			rev: CCTGTTTGGGAAGGAATATGA					

ABCC6	NM_001171	human	fwd: TTCTCTGTGTCGCTGGTGTC	309 bp	60°C	13 sec	87°C	-2.982
			rev: GGCACTGTGTATGGTGATGC					

MitATPase6	NC_001807	human	fwd: CTAAAGGACGAACCTGA	315 bp	55°C	13 sec	83°C	-3.608
			rev: TGGCCTGCAGTAATGTT					

HNF4α	NM_022180	rat	fwd: GCCTGCCTCAAAGCCATCAT	274 bp	55°C	11 sec	88°C	-3.697
			rev: GACCCTCCAAGCAGCATCTC					

HNF4αP1	D10554, EF193392	rat	fwd: AAATGTGCAGGTGTTGACCA	178 bp	60°C	7 sec	87°C	-3.296
			rev: CACGCTCCTCCTGAAGAATC					

HNF4αP2	AF329936	rat	fwd: CTCCAGTGGCGAGTCCTTAT	171 bp	60°C	7 sec	87°C	-2.273
	EF193390		rev: TCACGCTCCTCCTGAAGAAT					

MitATPase6	NC_001665	rat	fwd: CTAAAGGACGAACCTGA	315 bp	55°C	13 sec	83°C	-3.644
			rev: TGGCCTGCAGTAATGTT					

### Caco-2 cell culture

Caco-2 cells are a valuable source for HNF4α nuclear protein [21] and were obtained from and cultivated as recommended by DSMZ (Deutsche Sammlung von Mikroorganismen und Zellkulturen GmbH, Braunschweig, Germany) and seeded with a density of 4 × 10^6 ^cells per 75 cm^2 ^flask and harvested after 11 days of culture.

### Isolation of nuclear extracts

Nuclear extracts from Caco-2 cells were isolated by the modified method of Dignam et al [50]. Eleven days after seeding cells were washed twice with ice-cold PBS, scraped into microcentrifuge tubes and centrifuged for 5 min at 2000 × g, 4°C. Cell pellets were resuspended in lysis buffer (10 mM Tris pH 7.4, 2 mM MgCl_2_, 140 mM NaCl, 1 mM DTT, 4 mM Pefabloc, 1% Aprotinin, 40 mM β-glycerophosphate, 1 mM sodiumorthovanadate and 0.5% TX100) at 4°C for 10 min (300 μl for 1 × 10^7 ^cells), transferred onto one volume of 50% sucrose in lysis buffer and centrifuged at 14000 × g and 4°C for 10 min. Nuclei were resuspended in Dignam C buffer (20 mM Hepes pH 7.9, 25% glycerol, 420 mM NaCl, 1.5 mM MgCl_2_, 0.2 mM EDTA, 1 mM DTT, 4 mM Pefabloc, 1% Aprotinin, 40 mM β-glycerophosphate, 1 mM sodiumorthovanadate, 30 μl for 1 × 10^7 ^cells) and gently shaked at 4°C for 30 min. Nuclear debris was removed by centrifugation at 14000 × g at 4°C for 10 min. Protein concentrations were determined according to the method of Smith *et al *[51]. The extracts were aliquoted and stored at -70°C.

### Electrophoretic mobility shift assays

Forward and reverse oligonucleotides (for sequence information see Table 3) were purchased from MWG Biotech (Ebersberg/Muenchen, Germany), annealed and ^32^P-labeled using ^32^PγATP and T4-kinase (New England Biolabs, Frankfurt, Germany). 2,5 μg Caco-2 cell nuclear extract and 10^5 ^cpm (0.027 ng) radiolabeled probe were incubated in binding buffer consisted of 25 mM HEPES, pH 7.6, 5 mM MgCl_2_, 34 mM KCl, 2 mM DTT, 2 mM Pefabloc, 2% Aprotinin, 40 ng of poly (dI-dC)/μl and 100 ng of bovine serum albumin/μl for 20 minutes on ice. Free DNA and DNA-protein complexes were resolved on a 6% polyacrylamide gel (acrylamide: bisacrylamide ratio = 37.5:1). Super shift experiments were done with a 1 μg HNF4α specific antibody (sc-6556x, Santa Cruz Biotechnology, Heidelberg, Germany). DNA binding of nuclear extracts to the A-site of the *HNF1α*-promoter (HNF1pro) served as a positive control.

### Bioinformatic searches for HNF4α binding-sites

The transcription start site (TSS, +1) of the NCBI mRNA reference sequence (RefSeq) was aligned using the UCSC Genome Browser  for promoter annotation of the respective genes. Proximal promoters (up to -7000 bp) of human ABCB1, ABCB4 and ABCC1 to ABCC6 were checked for putative HNF4α binding-sites with the tool MATCH [52],  by employing two different weight matrix, i.e. V$HNF4_01, Transfac matrix (generated by Biobase) and V$zemlin13-11045 (self-generated). V$zemlin13-11045 is based on a collection of 33 well known HNF4α sites and was generated using the matrix generation tool of Biobase . At least one of both matrices has to exceed the cut-off of 0.9 for core similarity and 0.9 for matrix similarity (Table 2). Furthermore, proximal promoters (up to -7000 bp) of rat and mouse ABCB4 and ABCC1 were checked for putative HNF4α binding sites (Table 2).

### Immunohistochemistry

The sections were deparaffinized, demasked by heating, incubated with 0,6% H_2_O_2 _in methanol for 30 min, and subsequently with protein block serum-free reagent (Dako, Glostrup, Denmark) for 10 min. Incubation with polyclonal antibody (Santa Cruz Biotechnology, Heidelberg, Germany) against HNF4α (sc-6556x, 1:400 dilution for rat sections, 1:200 dilution for human sections) was performed for 45 min. The sections were rinsed with Tris-buffered saline, incubated with biotinylated universal secondary antibodies (Dako, Glostrup, Denmark) for 15 min and subsequently with horseradish peroxidase-conjugated streptavidin solution (Dako, Glostrup, Denmark) for 15 min. Labeling was detected using a diaminobenzidine (DAB) chromogen solution (Dako, Glostrup, Denmark) for 5 min. The sections were counterstained with hematoxylin before examination under light microscope. To confirm the specificity of the immunohistochemical localization, antibodies preabsorbed 2 h with a twenty fold excess of antigen for HNF4α (sc-6556P, Santa Cruz Biotechnology, Heidelberg, Germany) were used.

### siRNA silencing of HNF4α

Human HNF4α-specific siRNA probes were purchased from Qiagen (Hilden, Germany). Caco-2 cells (1,5 × 10^5 ^cells/well in 24-well plate) were transfected in triplicate for 48 h with 25 nM of the siRNA duplex using HiPerFect transfection reagent (Qiagen, Hilden, Germany). Alexa-Fluor488 labeld siRNA (Qiagen, Hilden Germany) was used as negative siRNA and as positive control for transfection efficiency. For transfection efficiency and stable expression of mitATPase6 after transfection see Niehof and Borlak, 2008 [22].

## Abbreviations

ABC transporter: ATP-binding cassette transporter; CSF: cerebrospinal fluid; DME: drug metabolizing enzymes; EMSA: electrophoretic mobility shift assay; HNF: hepatocyte nuclear factor; MDR: multidrug resistance gene family (ABCB); MRP: multidrug resistance related proteins gene family (ABCC); TTR: transthyretin.

## Authors' contributions

JB designed the entire study, supervised the experimental works and is responsible for the final writing of the manuscript, MN supervised the experiments and prepared the initial draft of the manuscript. Both authors read and approved the final manuscript.
